# One-year follow-up study after patients with severe COVID-19 received human umbilical cord mesenchymal stem cells treatment

**DOI:** 10.1186/s13287-022-02972-3

**Published:** 2022-07-16

**Authors:** Lei Shi, You Zheng, Zhi Cheng, Ningfei Ji, Changming Niu, Yan Wang, Tingrong Huang, Ruyou Li, Mao Huang, Xiaolin Chen, Lei Shu, Mingjing Wu, Kaili Deng, Jing Wei, Xueli Wang, Yang Cao, Jiaxin Yan, Ganzhu Feng

**Affiliations:** 1 Department of Pulmonary and Critical Medicine, the Second Affiliated Hospital of Nanjing Medical University, Nanjing, 210011 Jiangsu China; 2 Department of Nephrology, Huangshi Hospital of Traditional Chinese Medicine, Huangshi, 435000 Hubei China; 3 Department of Critical Care Medicine, the Second Affiliated Hospital of Nanjing Medical University, Nanjing, 210011 Jiangsu China; 4 Department of Respiratory and Critical Care Medicine, The First Affiliated Hospital of Nanjing Medical University, Nanjing, 210029 China; 5 Jiangsu Cell Tech Medical Research Institute, Nanjing, 211166 Jiangsu China; 6 Department of Respiratory Medicine, Huangshi Central Hospital, Huangshi, 435000 Hubei China; 7 Department of Pulmonary and Critical Care Medicine, the Second Affiliated Hospital of Nanjing Medical University, No. 121 Jiangjiayuan Rd, Gulou District, Nanjing, 210011 Jiangsu China; 8 Department of Respiratory Medicine, Sir Run Run Hospital, Nanjing Medical University, Nanjing, 211166, Jiangsu China

**Keywords:** Coronavirus disease 2019 (COVID-19), Human umbilical cord mesenchymal stem cells (h-UC-MSCs), 1-Year follow-up, Sequelae, Safety

## Abstract

**Background:**

The novel coronavirus is still mutating, and the pandemic continues. Meanwhile, many COVID-19 survivors have residual postinfection clinical manifestations. Human umbilical cord mesenchymal stem cells (hUC-MSCs) have been shown to be effective in the early stages of COVID-19.

**Objectives:**

The aim of this study was to investigate long-term safety and efficacy of treatment in patients with severe COVID-19 patients who had received hUC-MSCs therapy.

**Methods:**

Twenty-five discharged patients who had severe COVID-19 (including the standard treatment group and the standard treatment plus hUC-MSCs group) were enrolled in a 1-year follow-up. The assessment considered adverse effects (including effects on liver and kidney function, coagulation, ECG, tumor marker, and so on), pulmonary function, St George’s Respiratory Questionnaire (SGRQ), postinfection sequelae and serum concentration of Krebs von den Lungen-6 (KL-6), malondialdehyde (MDA), H_2_S, carnitine, and N-6 long-chain polyunsaturated fatty acids (N-6 LC-PUFAs).

**Measurements and main results:**

Pulmonary ventilation function had significantly improved at the 1-year follow-up in both the hUC-MSCs group and the control group compared with the 3-month follow-up (*P* < 0.01). Fatigue (60% [15/25]) remained the most common symptom at the 1-year follow-up. The rate of fatigue relief was significantly reduced in the hUC-MSCs group (25% [2/8]) compared to the control group (76.5% [13/17]) (*P* = 0.028). The level of KL-6 was significantly lower in the hUC-MSCs group (2585.5 ± 186.5 U/ml) than in the control group (3120.7 ± 158.3 U/ml) (*P* < 0.001). Compared with the control group, the hUC-MSCs group had a lower level of MDA (9.27 ± 0.54 vs. 9.91 ± 0.72 nmol/ml, *P* = 0.036). No obvious adverse effects were observed in the hUC-MSCs treatment group at 1 year after discharge.

**Conclusions:**

Intravenous transplantation of hUC-MSCs was a safe approach in the long term in the treatment of patients with severe COVID-19. In addition, hUC-MSCs had a positive effect on postinfection sequelae in COVID-19 survivors.

***Trial registration*:**

Chinese Clinical Trial Registration; ChiCTR2000031494; Registered 02 April 2020—Retrospectively registered, http://www.medresman.org

**Supplementary Information:**

The online version contains supplementary material available at 10.1186/s13287-022-02972-3.

## Introduction

Coronavirus disease 2019 (COVID-19), which is caused by the virus severe acute respiratory syndrome coronavirus 2 (SARS-CoV-2), developed rapidly into a global epidemics [1]. As of January 2022, more than 271.9 million confirmed cases of COVID-19, including 5.3 million deaths, have been reported to the WHO [2]. Several studies have reported that COVID-19 patients still have many postinfection clinical manifestations 1 year after discharge from the hospital, including dyspnea, fatigue, anxiety, impaired pulmonary function, chest CT abnormalities, and so on [3–5].

In a study of the SARS-CoV-2 Omicron variant in Denman, compared with Delta variant cases, the Omicron variant led to a higher rate of ICU admission (0.13% vs. 0.11%) [6]. At present, due to the limited efficacy of various antiviral agents in the short-term treatment of severe COVID-19 cases, the main treatment principles are still symptomatic and supportive therapy [7]. Then, an improvement in long-term sequelae is even less clear. An excessive inflammatory response is an important mechanism of disease aggravation and even death in patients with COVID-19 [8], and cytokine storms are closely related to clinical outcome in COVID-19 patients in the early stage of disease [9].

Many studies have shown that stem cells have immune modulation, tissue repair, and differentiation properties in infectious diseases [10–12]. In our previous study, we demonstrated the early-stage safety and preliminary therapeutic effect of hUC-MSCs in patients with severe COVID-19 [13, 14]. To date, there have been no 1-year follow-up studies on the safety and efficacy of stem cells therapy in severe COVID-19. The aim of this study was to further observe the long-term safety and improvement in sequelae of severe COVID-19 patients treated with hUC-MSCs.

## Methods

### Study design and participants

This was a longitudinal cohort study of patients with severe COVID-19 who were discharged from Huangshi Hospital of Traditional Chinese Medicine in Hubei Province from February 12 to March 25, 2020. The diagnosis criteria for severe COVID-19 followed a new coronavirus pneumonia diagnosis and treatment program (5th ed.) (in Chinese) [15]. The patients were randomly divided into 2 groups: a standard treatment group (control group) and a standard treatment plus human umbilical cord mesenchymal stem cells infusion group (hUC-MSCs group). In general, participation in this study was recommended for patients with severe COVID-19 cases whose clinical symptoms had not improved significantly after 7 to 10 days of standard treatment. The standard treatment was as follows: (1) supplemental oxygen (noninvasive or invasive ventilation); (2) antiviral agents (abidor/oseltamivir); (3) antibiotic agents (oral moxifloxacin or select antibiotics according to drug sensitivity tests); and (4) glucocorticoid therapy (1–2 mg/kg, less than a week).

The 1-year follow-up study was conducted from March 20 to April 14, 2021. This study enrolled 25 patients from our previously studied cohort who had been discharged 1 year ago (382–390 days) from Huangshi Hospital of Traditional Chinese Medicine in Hubei Province, China. The exclusion criteria included refusal to participate or loss of contact. None of the enrolled patients had been reinfected with SARS-CoV-2 in the past 1 year and developed other infectious diseases for nearly 2 weeks. The study was conducted in accordance with the Declaration of Helsinki and approved by the Ethics Committee of Huangshi Hospital of Traditional Chinese Medicine (No. HSZYPJ-2020-009-01). Written informed consent was obtained from all patients or their representatives who attended the follow-up visit.

### Cell preparation and transplantation

Clinical-grade hUC-MSCs were donated by the Jiangsu Cell Tech Medical Research Institute and Jiangsu Cell Tech Biotechnology Co of China. The product was registered at the China Clinical Trial Center (Registration No. ChiCTR2000031494). MSCs were prepared as previously described [13, 14]. Cells were cultured from the 2^nd^ passage to the 3^rd^ passage, which showed positive expression of CD73, CD90, and CD105 (> 95%) and negative expression of CD34, CD45, CD14 or CD11b, CD79α or CD19, and HLA-DR (< 2%) on the surface, as recommended by the International Society for Cellular Therapy (ISCT). The MSCs were suspended in 100 mL normal saline solution (0.9%), and the final number of transplanted cells was 2 × 10^6^ cells/kg. The hUC-MSCs were administered intravenously at a speed of 35 drops/min for approximately 1 h.

### Follow-up assessment

Eligible severe COVID-19 patients were invited to Huangshi Hospital of Traditional Chinese Medicine for two follow-up visits at 6 and 12 months after discharge. Follow-up procedures and indicators at 6 months are described in our previous studies [13, 14]. All patients were presented face to face with a series of questionnaires to assess their sequelae and quality of life. A self-reported symptom questionnaire was used to assess residual clinical symptoms 1 year later. The SGRQ was used to evaluate the impact of lung disease on patients' quality of life, and the questionnaire contained 50 items divided into three subgroups of symptoms, activities and effects [16]. Meanwhile, we performed a series of laboratory examinations to assess the patient's basic health status and adverse reactions, which included routine blood tests, biochemistry, blood gas, coagulation and SARS-COV-2 antibodies.

Pulmonary function testing was performed according to the standards of the American Thoracic Society, which include vital capacity (VC), forced vital capacity (FVC), forced expiratory volume in 1 s (FEV1), FEV1/FVC ratio, peak expiratory flow (PEF), and maximal voluntary ventilation (MVV). Salbutamol (a prebronchodilator) at 400 mg was administered during pulmonary function tests. Pulmonary function parameter results are shown as a percentage of the predicted value [17].

We tested the levels of plasma KL-6, MDA, H_2_S, carnitine, and N-6 LC-PUFA in all patients using ELISA kits. KL-6 is an important indicator for detecting pulmonary fibrosis, and MDA, H_2_S, carnitine, and N-6 LC-PUFA are key mediators that lead to fatigue symptoms with different mechanisms in patients.

### Outcome measures

The primary outcomes were adverse effects of MSCs therapy and long-term sequelae in patients with severe COVID-19. The secondary outcomes were SGRQ score, pulmonary function, and the levels of plasma KL-6, MDA, H_2_S, carnitine, and N-6 LC-PUFA in patients with severe COVID-19.

### Statistical analysis

All statistical analyses were performed with SPSS 21.0 software. Continuous variables were described using mean (± SD) or median (interquartile range, IQR) values, depending on whether they were normally distributed. Categorical variables were described as percentages. Continuous variables were compared using independent samples and related samples t tests, and categorical variables were compared using a *X*^*2*^ test. All statistical tests were two tailed, and a *P* value less than 0.05 was considered statistically significant.

## Results

### Follow-up procedure and baseline characteristics

**Fig. 1:**
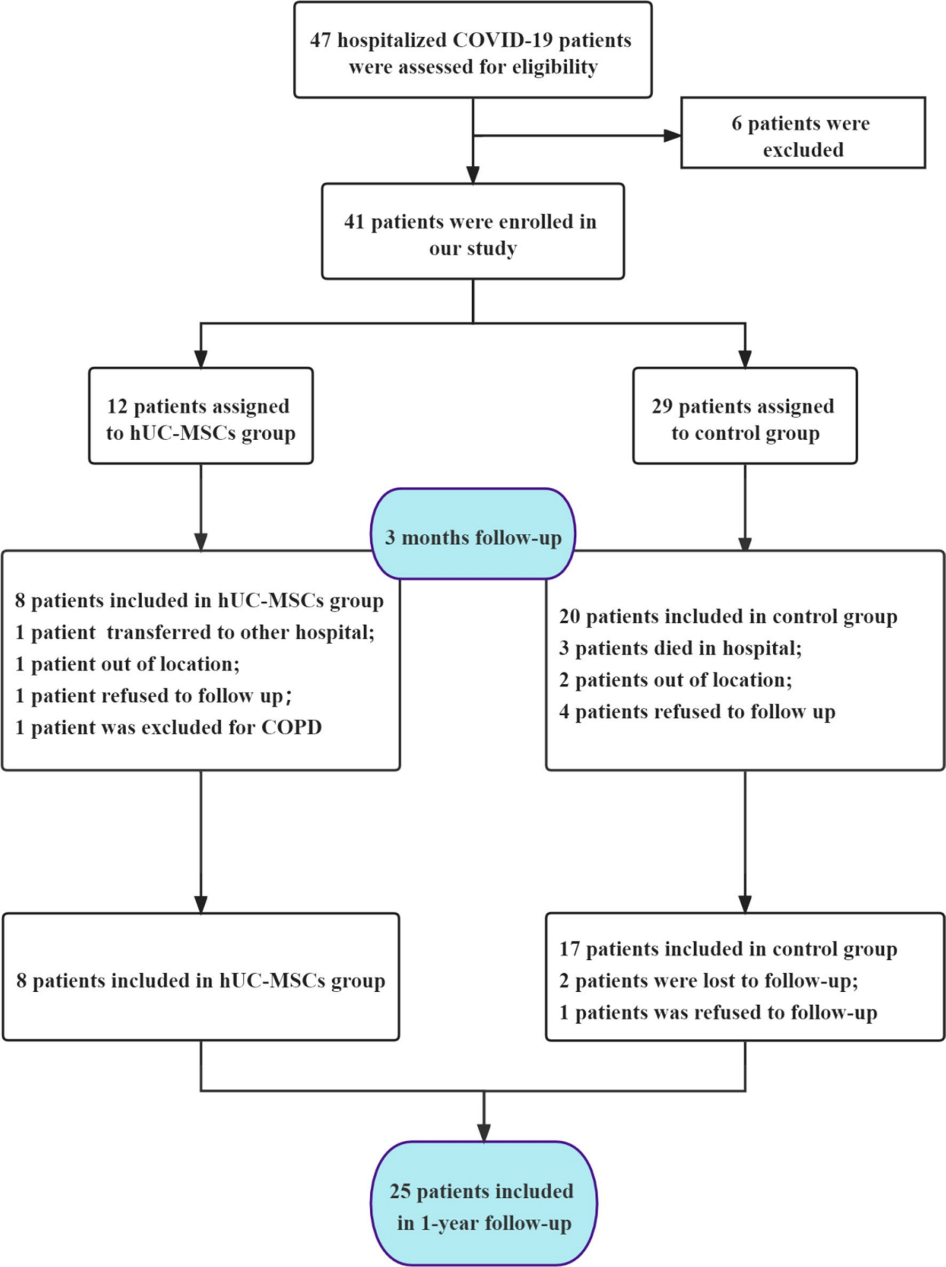
Flow diagram of the clinical trial for severe COVID-19 patients. Abbreviations: hUC-MSCs: human umbilical cord mesenchymal stem cells; COVID-19: coronavirus disease 2019

From February 12, 2020, to March 25, 2020, a total of 41 patients with severe COVID-19 were enrolled in this study, including 12 participants in the hUC-MSCs treatment group and 29 in the control group. At the 3-month follow-up, 7 cases were excluded (3 deaths occurred in the hospital and 4 cases could not be contacted), 5 cases refused follow-up, and 1 case was excluded from the study due to severe COPD. At the 1-year follow-up, 2 cases could not be contacted, and 1 case refused follow-up. Our final numbers included 8 cases enrolled in the hUC-MSCs treatment group and 17 cases in the control group (Fig. 1).

Table 1 shows the demographic characteristics and laboratory tests results for the 25 follow-up patients. The median age (interquartile range, IQR) was 51.00 (45.00, 67.00) years, with 50.50 (39.00, 72.75) years for the hUC-MSCs group and 52.00 (45.00, 63.00) years for the control group (*P* = 0.521). Forty-four percent (11/25) of patients were male (50.0% in the hUC-MSCs group vs. 41.18% in the control group, *P* = 1.000), and 28% (7/25) of patients smoked, including 37.5% (3/8) in the hUC-MSCs group and 23.53% (4/17) in the control group (*P* = 0.640). The BMIs in the hUC-MSCs treatment group and control group were 22.91 (20.40, 24.43) and 26.08 (21.51, 27.99), respectively (*P* = 0.181). Forty percent (10/25) of patients had comorbidities, including diabetes, hypertension, and other underlying diseases (50% in the hUC-MSCs treatment group vs. 35.29% in the control group, *P* = 0.667). In general, there were no significant differences in age, sex, smoking status, BMI, or comorbidities between the two groups.

**Table 1:** Demographics and characteristics of severe COVID-19 patients in 1-year follow-up

Variables	Follow-up after 1 year			
	Total patients (*n* = 25)	hUC-MSCs (*n* = 8)	Control (*n* = 17)	*P* value^#^
Age, years, median (IQR)	51.00 (45.00,67.00)	50.50 (39.00, 72.75)	52.00 (45.00,63.00)	0.521
Male	11 (44.00%)	4 (50.0%)	7 (41.18%)	1.000
Smokers	7 (28.00%)	3 (37.5%)	4 (23.53%)	0.640
Body mass index (BMI)	26.08 (22.16,27.99)	22.91 (20.40, 24.43)	26.08 (21.51,27.99)	0.181
Diabetes/hypertension	10 (40.00%)	4 (50.0%)	6 (35.29%)	0.667
*Blood routine examination*				
WBC, median (IQR), 10^9^ /L	5.60 (4.79,6.07)	5.62 (4.98,6.27)	5.60 (4.73,6.07)	0.813
NEU, 10^9^ /L	3.24 (2.65,3.84)	3.05 (2.24,4.15)	3.27 (2.65,3.76)	0.979
LYM, 10^9^ /L	1.76 (1.42,2.02)	1.77 (1.26,2.02)	1.76 (1.49,2.02)	0.543
MON, 10^9^ /L	0.31 (0.22,0.45)	0.26 (0.20,0.47)	0.31 (0.26,0.45)	0.776
HB, g/L	143.00 (130.00,156.00)	152.50 (128.75,162.50)	141.00 (130.00,152.50)	0.233
PLT, 10^9^ /L	227.00 (200.50,259.50)	243.00 (194.75,261.75)	226.00 (200.50,264.50)	0.745
*Blood biochemistry*				
AST, U/L	21.00 (16.50,25.00)	22.50 (15.00,32.00)	21.00 (18.00,23.50)	0.400
ALT, U/L	21.00 (14.00,30.00)	27.50 (19.25,36.25)	20.00 (13.50,24.00)	0.286
K, mmol/L	4.23 (3.98,4.47)	4.40 (4.27,4.77)	4.08 (3.92,4.37)	0.143
Na, mmol/L	142.10 (140.45,142.70)	141.05 (139.63,142.10)	142.30 (141.30,142.95)	0.141
Cl, mmol/L	107.30 (105.55,108.80)	106.40 (104.35,107.68)	108.00 (106.20,109.30)	0.135
BUN, mmol/L	4.26 (3.96,5.64)	4.07 (3.46,5.07)	5.19 (4.05,6.09)	0.132
Cr, μmol/L	66.00 (56.50,80.00)	60.50 (55.50,80.75)	67.00 (58.50,83.00)	0.484
*Myocardial injury markers*				
CK	107.00 (75.50,143.50)	99.00 (76.50,171.25)	107.00 (74.00,134.50)	0.786
CK-MB	1.83 (1.20,2.01)	1.72 (1.16,2.23)	1.83 (1.20,1.97)	0.384
LDH	175.00 (171.00,194.75)	172.00 (166.00,218.00)	176.00 (171.00,191.00)	0.238
*Blood coagulation*				
PT	10.50 (10.25,10.75)	10.50 (10.33,10.78)	10.50 (10.15,10.75)	0.316
APTT	32.90 (29.55,34.75)	33.45 (29.08,34.98)	32.60 (29.55,35.00)	0.926
D-Dimer	0.06 (0.04,0.12)	0.06 (0.04,0.26)	0.06 (0.04,0.12)	0.217
*Inflammatory markers*				
CRP	0.85 (0.61,1.72)	0.86 (0.58,2.80)	0.81 (0.63,1.39)	0.152
PCT	0.05 (0.05,0.05)	0.05 (0.05,0.05)	0.05 (0.05,0.05)	0.149

*hUC-MSCs* human umbilical cord mesenchymal stromal cells, *IQR* interquartile range, *WBC* white blood cell, *NEU* neutrophil, *LYM* lymphocyte, *Mon* monocyte, *PLT* platelet, *Hb* hemoglobin, *ALT* alanine aminotransferase, *AST* aspartate aminotransferase, *Cr* creatinine, *BUN* urea nitrogen, *PT* prothrombin time, *CK* creatine kinase, *CK-MB* creatine kinase-MB, *LDH* lactate dehydrogenase, *APTT* activated partial thromboplastin time, *CRP* C-reactive protein, *PCT* procalcitonin

^#^ hUC-MSCs group compared with control group

### Primary outcomes

One year after hUC-MSCs treatment, none of the patients showed abnormalities in liver function, routine blood tests, or ECGs. None of the patients developed significant skin pigmentation, blurred vision, neuropsychiatric abnormalities, or other serious complications. Two discharged patients had slightly elevated NSE and CA12-5, and one patient had a mild increase in creatinine (Table 2).

**Table 2:** Side effects of severe COVID-19 patients received hUC-MSCs

Patient number	Liver function		Urea	Cr	Blood routine	ECG	Tumor marker^#^	Thrombotic/embolic	Impaired vision	Analysis
	ALT	AST								
P1	25	37	4.96	60	Normal	Normal	Norma	Normal	Normal	
P2	29	23	6.14	82	Normal	Normal	CA12-5 elevated*	Normal	Normal	Aged patient
P3	33	39	3.92	77	Normal	Normal	Normal	Normal	Normal	
P4	15	18	3.44	57	Normal	Normal	Normal	Normal	Normal	
P5	11	34	3.50	50	Normal	Normal	NSE elevated*	Normal	Normal	Slightly elevated
P6	15	10	3.14	55	Normal	Normal	Normal	Normal	Normal	
P7	34	29	4.21	83	Normal	Normal	Normal	Normal	Normal	
P8	20	26	5.11	61	Normal	Normal	Normal	Normal	Normal	

Normal range: ALT 9-50U/L; AST 15-40U/L; Urea 1.7–8.3 mmol/L; Cr 40–80 umol/L; CA12-5: 0–35 U/ml; NSE: 0–16.3 ng/ml

*ECG* Electrocardiogram, *ALT* alanine aminotransferase, *AST* aspartate aminotransferase, *Cr* creatinine

^#^ Tumor marker includes: CEA, CA12-5, CA19-9, SCC, NSE, AFP, PSA (for males)

*CA12-5: 53 U/ml; NSE:18.5 ng/ml

At the 3-month follow-up, the most common symptoms (> 50%) were shortness of breath (76% [19/25]), fatigue (68% [17/25]), and sleep disorders (64% [16/25]). Fatigue (60% [15/25]) remained the most common symptom at the 1-year follow-up, whereas the rate of fatigue relief was significantly reduced in the hUC-MSCs group (25% [2/8]) compared to the control group (76.5% [13/17]) (*P* = 0.028). The proportion of patients with shortness of breath fell from 76% (19/25) at 3 months to 28% (7/25) at 1 year (*P* = 0.002), which also occurred in the hUC-MSCs treatment group (75% [6/8] vs. 12.5% [1/8], *P* = 0.041) and the control group (76.5% [13/17] vs. 35.3% [6/17], *P* = 0.037).

### Secondary outcomes

The SGRQ score decreased from 26.76 ± 11.34 at 3 months to 12.32 ± 8.88 at 1 year (*P* < 0.001) in both the hUC-MSCs treatment group (15.2 ± 3.69 vs. 9.13 ± 7.47, *P* = 0.012) and the control group (32.18 ± 9.46 vs. 13.82 ± 9.29, *P* < 0.001) (Table 3). The results of pulmonary function testing are shown in Table 4. Compared with the control group, there were no significant differences in the hUC-MSCs treatment group in terms of indicators of pulmonary function, including VC (% of predicted), FVC (% of predicted), FEV1 (% of predicted), FEV1/FVC, PEF (% of predicted), and MVV (% of predicted), at the 1-year follow-up. However, the above indicators significantly improved at the 1-year follow-up in both the hUC-MSCs group and the control group compared with 3 months after discharge. The level of KL-6 was significantly lower in the hUC-MSCs group (2585.5 ± 186.5 U/ml) than in the control group (3120.7 ± 158.3 U/ml) (*P* < 0.001). Compared with the control group, the hUC-MSCs group had a lower level of MDA (9.27 ± 0.54 vs. 9.91 ± 0.72 nmol/ml, *P* = 0.036) and a higher level of N-6 LC-PUFAs (200.1 ± 11.6 vs. 209.1 ± 11.3 pg/ml, *P* = 0.083). There were no significant differences in the levels of H_2_S and carnitine.

**Table 3:** Long-term sequelae and SGRQ score of severe COVID-19 patients were followed at 3 months and 1 year

Syndrome	Total patients (*n* = 25)			hUC-MSC (*n* = 8)			Control (*n* = 17)			1-year follow-up		
	3 Months	1 Year	*P* value^#^	3 Months	1 Year	*P* value^#^	3 Months	1 Year	*P* value^#^	Control	hUC-MSC	*P* value^##^
Fatigue	17(68.0%)	15(60.0%)	0.769	4(50.0%)	2(25.0%)	0.608	13(76.5%)	13(76.5%)	1.000	13(76.5%)	2(25.0%)	0.028*
Short of breath	19(76.0%)	7(28.0%)	0.002**	6(75.0%)	1(12.5%)	0.041*	13(76.5%)	6(35.3%)	0.037*	6(35.3%)	1(12.5%)	0.362
Sleep disorders	16(64.0%)	12(48.0%)	0.393	5(62.5%)	3(37.5%)	0.619	11(64.7%)	9(52.9%)	0.728	9(52.9%)	3(37.5%)	0.642
Cough	9(36.0%)	3(12.0%)	0.095	3(37.5%)	1(12.5%)	0.569	6(35.3%)	2(11.8%)	0.225	2(11.8%)	1(12.5%)	1.000
SGRQ (Mean ± SD)	26.76 ± 11.34	12.32 ± 8.88	0.000***	15.25 ± 3.69	9.13 ± 7.47	0.012*	32.18 ± 9.46	13.82 ± 9.29	0.000***	13.82 ± 9.29	9.13 ± 7.47	0.224

*hUC-MSCs* human umbilical cord mesenchymal stem cells, *SGRQ* St George’s Respiratory Questionnaire, ^#^ 3 months compared with 1 year, ^##^ hUC-MSCs group compared with control group

*P < 0.05; ** *P* < 0.01; ***P < 0.001

**Table 4:** Results of lung function test in severe COVID-19 patients were followed at 3 months and 1 year

Parameter	Total patients (*n* = 25)			hUC-MSC (*n* = 8)			Control (*n* = 17)			1-year follow-up		
	3 Months	1 Year	*P* value	3 Months	1 Year	*P* value	3 Months	1 Year	*P* value	Control	hUC-MSC	*P* value
*VC*												
VC (Mean ± SD)	2.91 ± 0.74	3.3 ± 0.81	0.055	2.72 ± 0.88	3.14 ± 0.93	0.102	3 ± 0.67	3.35 ± 0.84	0.144	3.35 ± 0.84	3.14 ± 0.93	0.575
VC (% of predicted)	81.4 ± 14.21	101.68 ± 15.84	0.000***	72.63 ± 9.86	99.75 ± 12.99	0.002**	85.53 ± 14.27	102.59 ± 17.31	0.003**	102.59 ± 17.31	99.75 ± 12.99	0.685
*N*, ≤ 80% predicted	11(44.0%)	1(4.0%)	0.001**	5(62.5%)	0(0%)	0.026*	6(35.3%)	1(6.9%)	0.085	1(6.9%)	0(0%)	1.000
*FVC*												
FVC (Mean ± SD)	2.83 ± 0.75	3.02 ± 0.78	0.182	2.68 ± 0.89	2.8 ± 0.79	0.507	2.89 ± 0.7	3.12 ± 0.78	0.257	3.12 ± 0.78	2.8 ± 0.79	0.350
FVC (% of predicted)	80.04 ± 14.35	93.24 ± 11.27	0.000***	72.63 ± 9.46	89.88 ± 9	0.003**	83.53 ± 15.14	94.82 ± 12.12	0.022*	94.82 ± 12.12	89.88 ± 9	0.316
*N*, ≤ 80% predicted	13(52.0%)	3(12.0%)	0.002**	6 (75.0%)	0(0%)	0.007**	7(41.2%)	3(17.6%)	0.259	3(17.6%)	0(0%)	0.527
*FEV1*												
FEV1 (Mean ± SD)	1.89 ± 0.71	2.54 ± 0.76	0.001**	2.12 ± 0.64	2.37 ± 0.73	0.193	1.78 ± 0.73	2.62 ± 0.78	0.002**	2.62 ± 0.78	2.37 ± 0.73	0.459
FEV1 (% of predicted)	65.84 ± 23.94	90.72 ± 10.97	0.000***	71.88 ± 8.46	89.13 ± 6.85	0.001**	63 ± 28.31	91.47 ± 12.57	0.001**	91.47 ± 12.57	89.13 ± 6.85	0.628
≤ 80% predicted	18(72.0%)	4(16.0%)	0.000 ***	6(75.0%)	0(0%)	0.007**	12(70.6%)	4(23.5%)	0.015*	4(23.5%)	0(0%)	0.283
*FEV1/FVC (%)*	0.67 ± 0.19	0.84 ± 0.07	0.000***	0.8 ± 0.08	0.84 ± 0.06	0.186	0.61 ± 0.2	0.83 ± 0.08	0.000	0.83 ± 0.08	0.84 ± 0.06	0.829
≤ 70%	12(48.0%)	1(4.0%)	0.000***	1(12.5%)	0(0%)	1.000	10(58.8%)	1(6.9%)	0.000	1(6.9%)	0(0%)	1.000
*PEF*	2.82 ± 1.83	4.44 ± 1.42	0.001	3.59 ± 1.81	4.17 ± 1.73	0.391	2.46 ± 1.78	4.58 ± 1.28	0.001	4.58 ± 1.28	4.17 ± 1.73	0.514
PEF (% of predicted)	38.4 ± 27.05	65.92 ± 18.8	0.000	45.5 ± 22.87	62.63 ± 20.03	0.034	35.06 ± 28.84	67.47 ± 18.62	0.001	67.47 ± 18.62	62.63 ± 20.03	0.559
*MVV*	70 ± 26.49	90.44 ± 25.23	0.005	71.56 ± 39.61	89.44 ± 35.4	0.241	69.26 ± 19.08	90.91 ± 20.14	0.011	90.91 ± 20.14	89.44 ± 35.4	0.895
MVV (% of predicted)	65.32 ± 15.52	86.74 ± 15.94	0.000	67.13 ± 20.16	85.31 ± 17.62	0.014	64.47 ± 13.46	87.41 ± 15.6	0.000	87.41 ± 15.6	85.31 ± 17.62	0.766

*VC* vital capacity, *FVC* vital capacity, *FEV1* forced expiratory volume in 1 s, *PEF* peak expiratory flow, *MVV* maximal voluntary ventilation

*P < 0.05; **P < 0.01; ***P < 0.001

## Discussion

Despite efforts to strengthen vaccination, quarantine policies, and restrictions on social distancing, the number of confirmed cases and deaths of COVID-19 patients around the world continues to rise rapidly [2]. In addition, patients with severe COVID-19 often develop ARDS, and the prognosis is frequently poor [18, 19]. Several studies have shown that nearly half of COVID-19 survivors still have at least one clinical sequelae at the 1-year follow-up [3, 5].

Currently, there are several methods to treat severe COVID-19 patients, such as MSCs-based therapy, convalescent plasma, antiviral drugs, Chinese traditional medicine, and so on. In a series of our previous studies and in many other reports, we demonstrated the safety and short-term efficacy of stem cells therapy. In the acute phase of COVID-19, hUC-MSCs may inhibit an excessive inflammatory response through their immunomodulatory properties. In the convalescence phase, MSCs may participate in the tissue repair of alveolar epithelial cells through their strong differentiation abilities [13, 14, 20–22]. However, the long-term efficacy and safety of stem cells in the treatment of COVID-19 are still unclear. To our knowledge, there have been no 1-year follow-up studies for COVID-19 patients who received stem cells therapy.

Based on previous studies on the safety and initial efficacy of stem cells in the treatment of COVID-19, we conducted a 1-year follow-up study to explore the long-term safety and efficacy of stem cells. Twenty-five patients, including a control group and an hUC-MSCs treatment group, had almost normal ranges in terms of routine blood tests, liver and kidney function, coagulation, myocardial injury, and inflammatory markers. Meanwhile, the above indicators were not significantly different between the two groups. In previous short-term follow-up studies, no serious adverse events were observed after 1–3 months of stem cells therapy in patients with COVID-19 [20, 22, 23]. In addition, a 5-year follow-up study of stem cells treatment for H7N9 did not reveal any adverse effects [24]. In this study, we observed a slight elevation in tumor markers, including CA12-5 and NSE, in patients 2 and 5, respectively. Patient 2 already had a mild elevation of CA12-5 at the 3-month follow-up and continued to show a similar elevation of CA12-5 at the 1-year follow-up. Although a slight NSE elevation was found in patient 5, we did not detect any evidence of tumors. Patient 7 had a mild increase in creatinine from 72 to 83 (normal < 80) over 9 months. In addition, no adverse effects, such as significant skin pigmentation, blurred vision, or neuropsychiatric abnormalities, were observed in any patients, which confirmed the safety of hUC-MSCs therapy for COVID-19 at the 1-year follow-up.

**Fig. 2:**
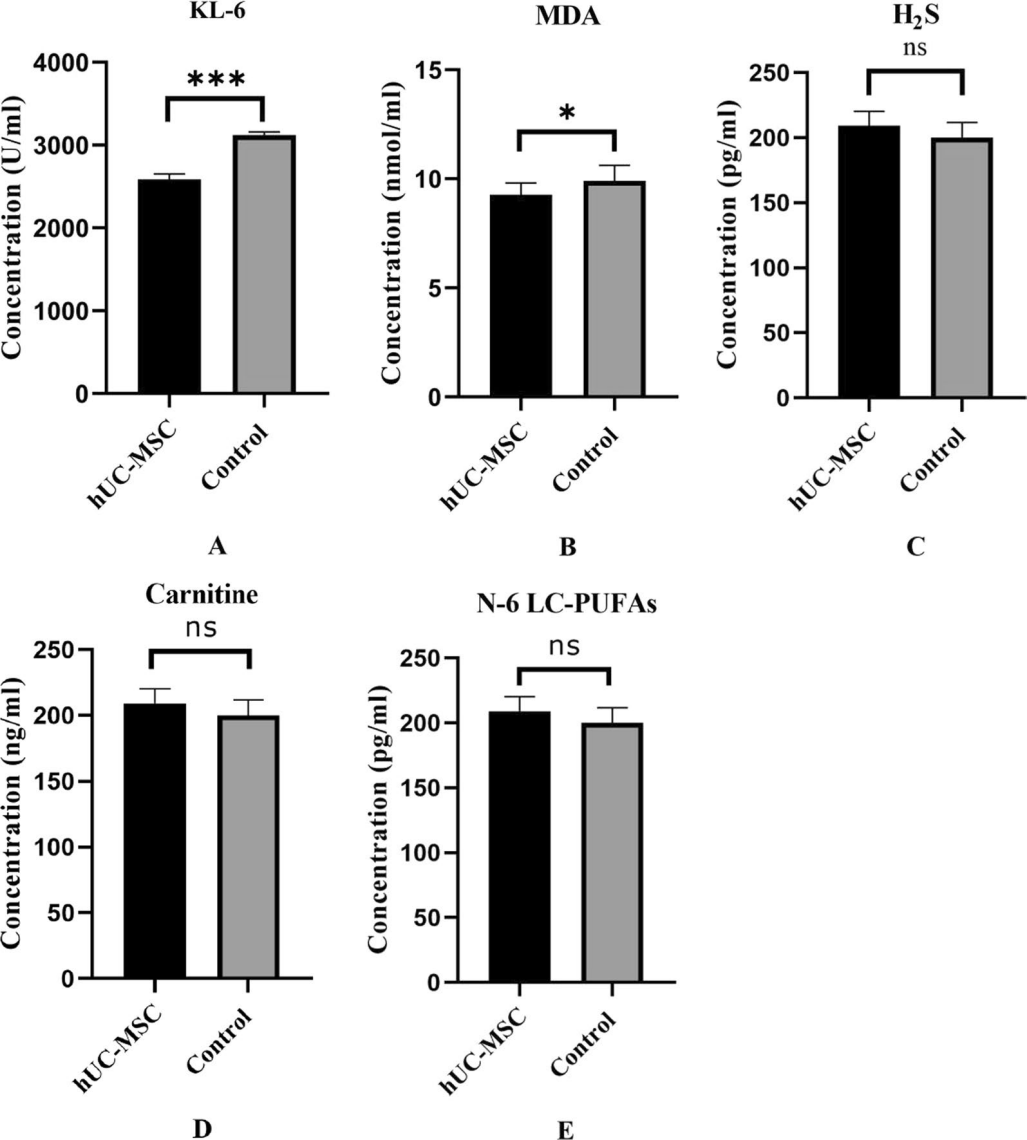
The levels of plasma KL-6, MDA, H_2_S, carnitine, and N-6 LC-PUFA in severe COVID-19 patients of hUC-MSCs and control group. **A** The level of KL-6 was significantly lower in hUC-MSCs group than that of control group (2585.5 ± 186.5 l vs. 3120.7 ± 158.3 U/ml, *P* < 0.001); **B** The hUC-MSC group had a lower level of MDA compared with control group (9.2 ± 0.54 vs. 9.91 ± 0.72 nmol/ml, *P* = 0.0361); **C** There was no significant difference in the level of H_2_S (60.19 ± 2.86 vs. 59.62 ± 2.17, *P* = 0.58); **D** There was no significant difference in the level of Carnitine (40.73 ± 5.01 vs. 43.100 ± 2.45, *P* = 0.122); **E** The hUC-MSC group had a higher level of N-6 LC-PUFAs compared with control group (200.1 ± 11.6 vs. 209.1 ± 11.3 pg/ml, *P* = 0.083). *, *P* < 0.05; **, *P* < 0.01; ***, *P* < 0.001. Abbreviations: MDA: malondialdehyde; N-6 LC-PUFAs: N-6 long-chain polyunsaturated fatty acids; KL-6: Krebs von den Lungen-6; hUC-MSCs: human umbilical cord mesenchymal stem cells

In our study, we found that almost none of patients had obvious lesions or fibrous band shadows at 1-year follow-up (Additional file 1: Figure S1). Unfortunately, because some patients refused chest CT examination, we only analyzed the chest CT images of 17 of the 25 patients (7 in the MSCs group and 10 in the control group). Meanwhile, we found that pulmonary ventilation parameters in the vast majority of patients with severe COVID-19 were within the normal range at the 1-year follow-up, which was similar to other 1-year follow-up studies [3, 25]. We also found significant improvement in pulmonary function indicators at the 1-year follow-up compared with the 3-month follow-up, such as VC (% of predicted), FVC (% of predicted), FEV1 (% of predicted), FEV1/FVC, PEF (% of predicted), and MVV (% of predicted), which showed that pulmonary ventilation function in most severe COVID-19 patients had basically returned to normal by 1 year after discharge. In this regard, there were no significant differences in pulmonary ventilation function between the hUC-MSCs group and the control group at the 1-year follow-up. However, in the preceding series of 1-year follow-up studies, patients with severe COVID-19 had varying degrees of impairment in lung diffusion function, ranging from 31 to 38% [3, 25]. Follow-up studies of SARS have also shown that lung diffusion function impairment can last for months or even years [26–28]. Unfortunately, due to the limited conditions of primary hospitals, we were unable to conduct pulmonary diffusion function tests. KL-6, a predictive marker of interstitial lung disease, reflects the extent of damage to alveolar type II epithelial cells [29, 30], which has also been proven to be effective in predicting the prognosis of COVID-19 patients [31, 32]. Zeng et al. conducted a study on the proteomics of bronchoalveolar lavage fluid and showed a significant decrease in KL-6 in the lavage fluid of patients with severe COVID-19 compared with non-COVID-19 patients [33]. Our study found that KL-6 in the hUC-MSCs group was significantly lower than in the control group (2585.53 ± 186.45 vs. 3120.69 ± 158.34, *P* < 0.001) (Fig. 2A), indicating that hUC-MSCs may improve lung diffusion function by promoting alveolar epithelial cell regeneration.

In our previous study, we found that shortness of breath was the most common symptom of patients with severe COVID-19 at 3 months after discharge (Table 4). However, the most common sequelae shifted to fatigue at the 1-year follow-up, similar to other follow-up studies [3, 25, 34]. Lam MH et al. also showed that fatigue was the most common sequelae in SARS patients, even lasting for up to 4 years [35]. Interestingly, the rate of fatigue in the hUC-MSCs group was significantly lower than that in the control group (25.0% vs. 76.5%, *P* = 0.028). The causes and mechanisms of fatigue in COVID-19 survivors are unclear but based on previous studies of patients with chronic fatigue, causes may include lung diffusion function, redox imbalance, and impaired mitochondrial function [36–39]. Hence, we assessed MDA, H_2_S, carnitine, and N-6 LC-PUFAs, representing lipid peroxidation, protein sulfhydration, mitochondrial function, and the function of cell membranes, respectively [36–41]. We found that the levels of MDA significantly decreased in the hUC-MSCs group compared with the control group. MDA is a metabolite product when oxygen free radicals attack fatty acids on the cell membrane, directly reflecting the degree of lipid peroxidation. There was a direct positive correlation between MDA levels and fatigue symptoms [42]. Moreover, stem cells can reduce MDA production by regulating oxygen free radicals and inflammation [43, 44]. Therefore, we speculated that hUC-MSCs reduced MDA production by regulating oxidative stress, thereby improving fatigue symptoms in COVID-19 patients. At the same time, we detected a high level of N-6 LC-PUFAs in the hUC-MSCs group, although there was no significant difference, which may be due to an insufficient sample size. Viral infection may impair the biosynthesis of N-6 long-chain polyunsaturated fatty acids by inhibiting δ-6 desaturation of the essential fatty acids, thereby impairing cell membrane function and leading to fatigue symptoms [40]. In general, hUC-MSCs may alleviate fatigue in COVID-19 patients in a variety of ways, and further research is needed.

Our study has several limitations. First, this is a single-center and small-sample longitudinal cohort study, so systemic bias is inevitable. Second, due to a lack of equipment at the primary hospital, lung diffusion function testing was not performed. Third, this is a preliminary study on stem cells therapy for severe COVID-19, and the specific mechanism still needs further research.

## Conclusions

In our 1-year follow-up, hUC-MSCs therapy remained a safe and effective means to combat severe COVID-19 infection. In addition, hUC-MSCs significantly alleviated fatigue symptoms in COVID-19 patients, possibly by reducing MDA production.

## Supplementary Information

**Additional file 1.** Almost none of patients had obvious lesions or fibrous band shadows at 1-year follow-up.

## Data Availability

The datasets used and/or analyzed in this study are available from the corresponding author upon reasonable request.

## Abbreviations

COVID-19: Coronavirus disease 2019
hUC-MSCs: Human umbilical cord mesenchymal stem cells
SGRQ: St George’s Respiratory Questionnaire
CRP: C-reactive protein
IQR: Interquartile range
CK: Creatine kinase
LDH: Lactate dehydrogenase
ALT: Alanine aminotransferase
AST: Aspartate aminotransferase
VC: Vital capacity
FVC: Vital capacity
FEV1: Forced expiratory volume in 1s
PEF: Peak expiratory flow
MVV: Maximal voluntary ventilation
MDA: Malondialdehyde
N-6 LC-PUFAs: N-6 long-chain polyunsaturated fatty acids
KL-6: Krebs von den Lungen-6
