# Supplementary material for: One-year follow-up study after patients with severe COVID-19 received human umbilical cord mesenchymal stem cells treatment
Source: Stem Cell Res Ther. 2022 Jul 16;13:321. doi: 10.1186/s13287-022-02972-3 (PMC9288258; doi:10.1186/s13287-022-02972-3)
Supplement: Supplementary file 1 — Additional file 1. Almost none of patients had obvious lesions or fibrous band shadows at 1-year follow-up. [file 13287_2022_2972_MOESM1_ESM.docx]

**Supplementary Figure 1.** Chest computerized tomography (CT) images in severe COVID-19 patients of hUC-MSC and control groups.


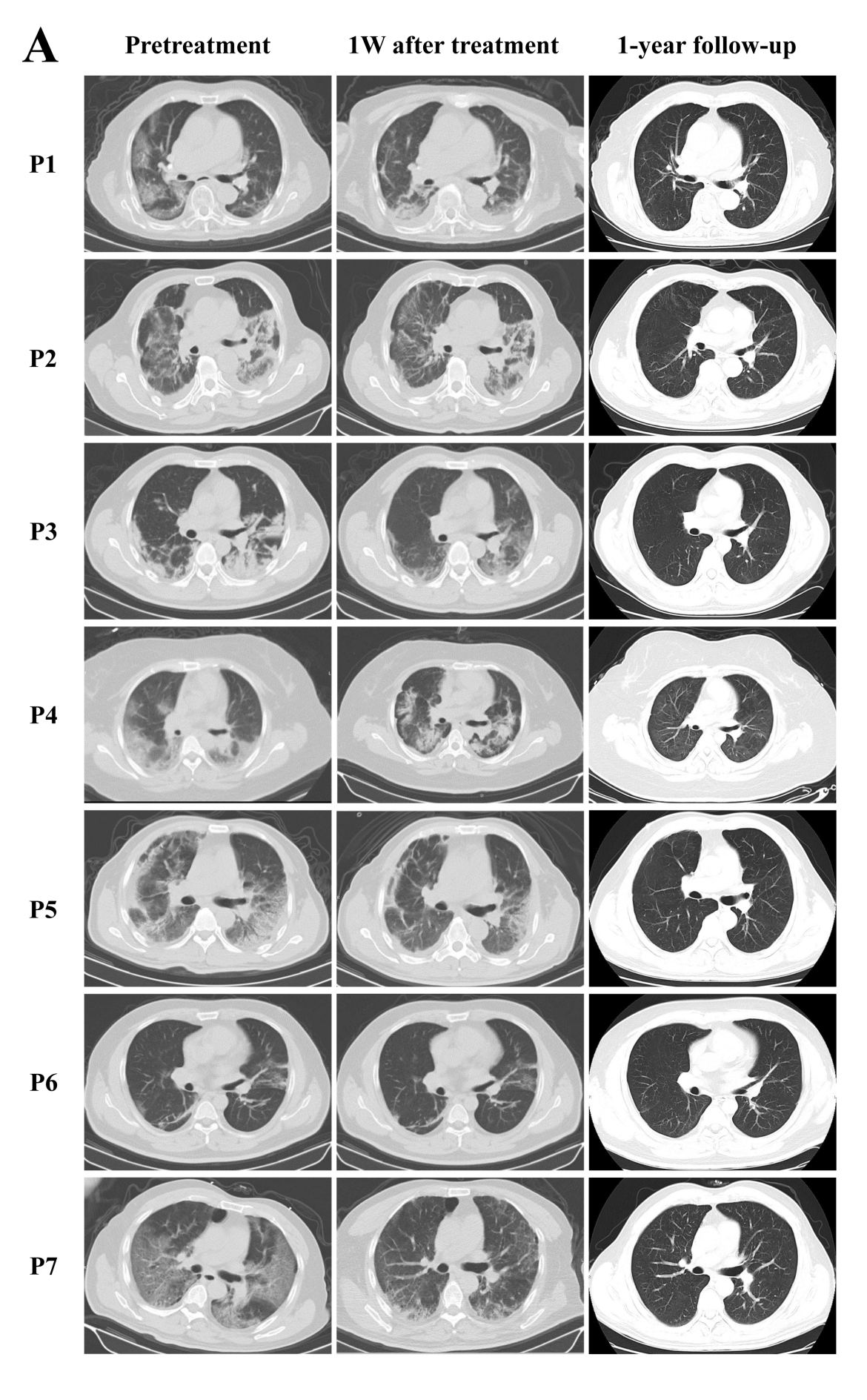


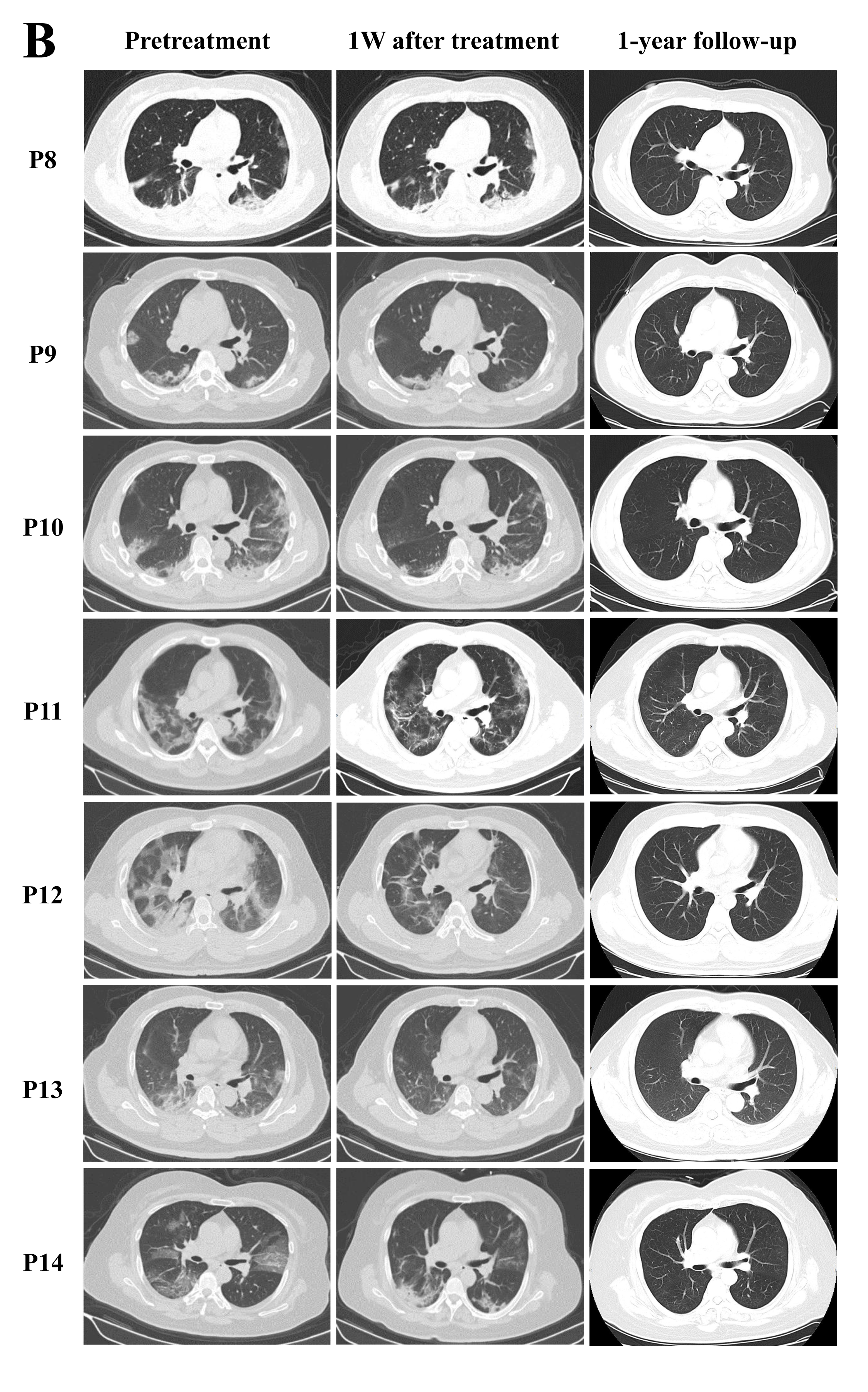


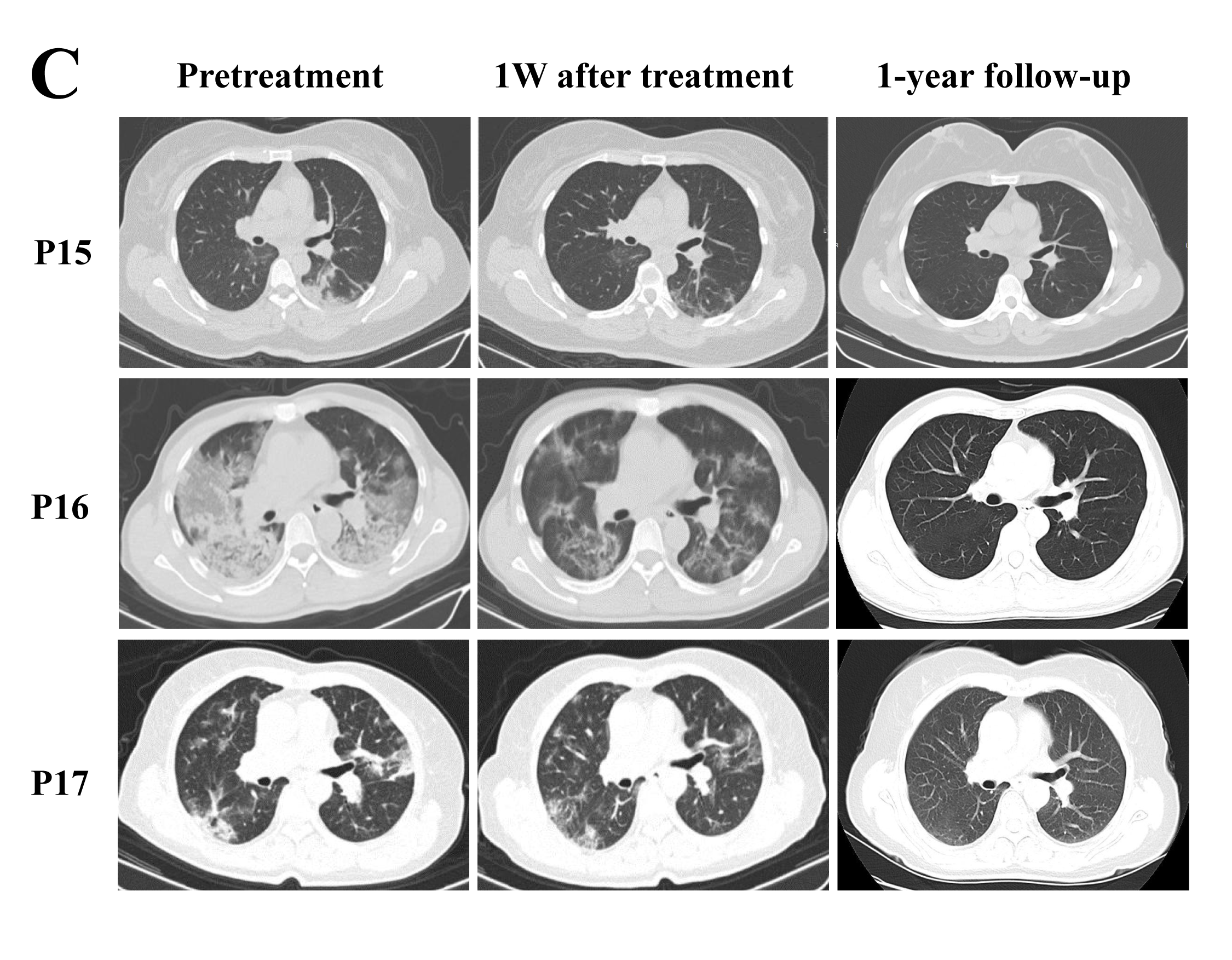
Figure 1A: hUC-MSCs group (P1 to P7 were the patients from the hUC-MSCs group)； Figure 1B and Figure 1C: Control group (P8 to P17 were patients from the control group. )；

Almost none of patients had obvious lesions or fibrous band shadows at 1-year follow-up.
